# Metabolomic Profiles and Differential Constituents of *Andrographis paniculata* (Burm. f.) in Different Growth Stages and Parts

**DOI:** 10.3390/molecules30071490

**Published:** 2025-03-27

**Authors:** Enming Hu, Rui Cheng, Annian Liu, Ya Wang, Huali Long, Jinjun Hou, Daoping Wang, Wanying Wu, Xingdong Wu

**Affiliations:** 1 State Key Laboratory of Discovery and Utilization of Functional Components in Traditional Chinese Medicine, Guiyang 550014, China; 47780huhanqing@gmail.com (E.H.); wangdaoping@gmc.edu.cn (D.W.); 2 Guizhou Engineering Research Center of Industrial Key-Technology for Dendrobium Nobile, Joint International Research Laboratory of Ethnomedicine of Ministry of Education, Zunyi Medical University, Zunyi 563000, China; 18636703714@163.com (R.C.); liuan_0808@163.com (A.L.); 18275687211@163.com (Y.W.); 3 Natural Products Research Center of Guizhou Province, Guiyang 550014, China; 4 National Engineering Research Center of TCM Standardization Technology, Shanghai Institute of Materia Medica, Chinese Academy of Sciences, Shanghai 201203, China; longhuali@simm.ac.cn (H.L.); jinjun_hou@simm.ac.cn (J.H.)

**Keywords:** *Andrographis paniculata*, different plant parts, different growth stages, non-targeted metabolomics, quality markers

## Abstract

*Andrographis paniculata* (Burm. f.) and its products have a long history of medicinal use in Asia. *A. paniculata* products are mainly made from the root extraction of stems, leaves and parts, but there may be differences in the proportion of different parts and different harvest times, which ultimately leads to certain differences in product quality. In this study, the chemical components and non-targeted metabolomics were characterized, and the characteristic compounds in different parts of *A. paniculata* at various growth stages were analyzed. By utilizing polygonal mass defect filtering, precursor ion lists, and a self-built compound library, a total of 225 components were identified in *A. paniculata*. Notably, spermidine derivatives and phosphatidylcholines were reported for the first time in this plant species. In total, 41 differential components were identified in different parts of *A. paniculata*. These findings provide scientific evidence for the selection of quality markers in *A. paniculata* and its products.

## 1. Introduction

*Andrographis paniculata* (Burm. f.), a member of the *genus I* in the Acanthaceae family, encompasses three species: *A. paniculata*, *A. laxiflora*, and *A.* var. *glomerulifera* [1]. It predominantly thrives in subtropical regions of China, India, Thailand, and other Asian countries [2,3]. *A. paniculata* is renowned for its abundance of diterpene lactones, flavonoids, and organic acids, and has been traditionally employed in Asian traditional medicine for the treatment of ailments such as cardiovascular diseases, snakebites, and malaria [2,4,5]. Its exceptional therapeutic efficacy has garnered recognition in major pharmacopoeias worldwide, including the Chinese Pharmacopoeia, United States Pharmacopeia, and European Pharmacopoeia. Presently, several *A. paniculata*-derived products have been developed, including tablets, capsules, and formulations based on the active diterpene lactones [6]. Andrographolide, dehydroandrographolide, 14-deoxyandrographolide, and neoandrographolide are the four major diterpene lactones commonly employed to assess the quality of *A. paniculata* and its related products [7]. However, our previous research conducted by our group involved a random examination of four diterpene lactones in *A. paniculata* tablets from ten different manufacturers in China, revealing significant variations in andrographolide content among the samples (Figure S1, Table S1). These variations may be attributed to differences in the proportion of roots and stems utilized as raw materials, as well as discrepancies in the harvesting seasons of the herbal ingredients [8]. Thus, relying solely on the four major diterpene lactones for the quality evaluation of *A. paniculata* and its formulations is insufficient. Consequently, it is imperative to systematically compare and analyze the chemical constituents of *A. paniculata* collected at different growth stages and from various plant parts to identify additional quality markers and determine the optimal harvesting period.

Plants exhibit remarkable plasticity in adapting to diverse growth stages and environmental conditions by producing a wide array of secondary metabolites, which play crucial roles in reproduction and self-protection [9,10]. Consequently, substantial variations in the chemical composition of plants are often observed across different growth stages and environmental contexts [11]. While previous studies have reported changes in the content of andrographolide, dehydroandrographolide, and neoandrographolide in *A. paniculata* at different growth stages, investigations into the differential chemical constituents among distinct growth stages and plant parts of *A. paniculata* remain scarce [12]. Non-targeted metabolomics, a comprehensive approach that encompasses the global analysis of metabolites, offers a valuable means to study the relative changes in metabolite types and quantities within biological systems under physiological and pathological conditions [13]. Notably, untargeted metabolomics has proven successful in identifying disease biomarkers and screening quality markers in the field of herbal medicine [14,15]. Moreover, the advent of high-resolution mass spectrometry has facilitated the high-throughput characterization of chemical constituents within complex systems, thanks to its exceptional precision, sensitivity, and resolution [16,17]. Consequently, a synergistic integration of high-resolution mass spectrometry and untargeted metabolomics can be employed to discern and compare the chemical composition of different parts of *A. paniculata* at various growth stages.

In this study, we aimed to comprehensively characterize the chemical constituents of *A. paniculata* based on samples collected from different growth stages and plant parts within the cultivation area. Firstly, we employed UHPLC-LTQ-Orbitrap-MS^n^ technology in conjunction with a self-built database for the systematic characterization of *A. paniculata*. Furthermore, untargeted metabolomics analysis was conducted to identify differential constituents among different growth stages and parts of *A. paniculata*. These analyses aimed to elucidate the composition of chemical constituents in *A. paniculata*, providing insights into the determination of optimal harvesting periods and contributing to the selection of quality markers for *A. paniculata* and its derived products.

## 2. Experiment

### 2.1. Reagents and Materials

**Figure 1:**
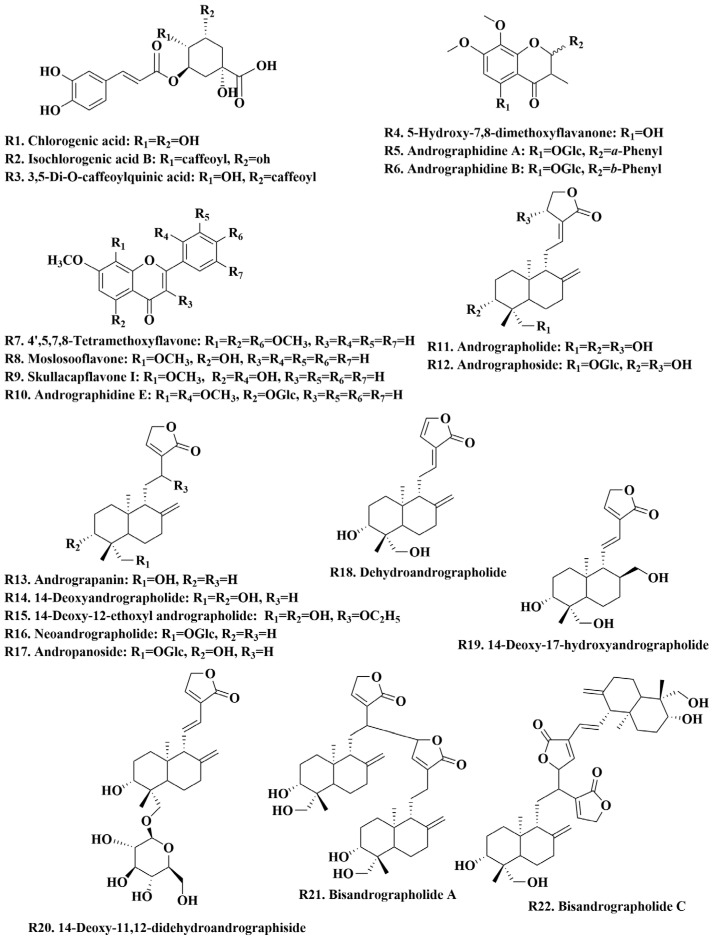
Chemical structure of 22 reference standards.

A total of 22 chemical reference standards were used for the characterization of chemical constituents, including andrographidine A (R5)/B (R6)/E (R10), 5-hydroxy-7,8-dimethoxyflavanone (R4), 4′,5,7,8-tetramethoxyflavone (R7), moslosooflavone (R8), skullcapflavone I (R9), chlorogenic acid (R1), isochlorogenic acid B (R2), 3,5-di-O-caffeoylquinic acid (R3), andrographolide (R11), dehydroandrographolide (R18), 14-deoxyandrographolide (R14), neoandrographolide (R16), andrographoside (R12), andropanoside (R17), 14-Deoxy-11,12-didehydroandrographiside (R20), andrograpanin (R13), 14-deoxy-17-hydroxyandrographolide (R19), 14-deoxy-12-ethoxyl andrographolide (R15), and bisandrographolide A (R21)/C (R22). These reference standards were purchased from Shanghai Natural-Standard Company (Shanghai, China), Sichuan Vikki Biotechnology Co., Ltd. (Chengdu, China), and BioBioPha Co., Ltd. (Kunming, China). The purity of these reference standards was more than 95% after detection by HPLC. These standards included seven flavonoids, three organic acids, and twelve diterpene lactones, and their chemical structures are shown in Figure 1. HPLC-grade and MS-grade acetonitrile were obtained from Merck (Darmstadt, Germany), and MS-grade formic acid was purchased from Roe Scientific Inc. (Newark, NJ, USA). All other reagents used in this study were of analytical grade. Ultra-pure water was prepared in the laboratory. *A. paniculata* samples at different growth stages and plant parts were provided by medicinal plant growers in Guigang, Guangxi province, China (Table S2) and authenticated as plants of *A. paniculata* in Lamiaceae family by Professor Guo Dean. Voucher specimens of *A. paniculata* were deposited in the Center for Modernization of Traditional Chinese Medicine, Shanghai Institute of Materia Medica, Chinese Academy of Sciences.

### 2.2. Preparation of Reference and Sample Solutions

Accurate amounts of the 22 reference standards were weighed. *A. paniculata* sample was ground to 60 mesh after drying under natural conditions, 0.5 g of each sample was accurately weighed, and sonicated with 25 mL of 75% methanol for 30 min (100 W, 35 kHz). After the extraction solution has cooled to room temperature, the lost weight is replenished with 75% methanol. After shaking, centrifuge at 12,000 rpm for 10 min and filter through a 50 μm Millipore filter. Weigh the appropriate amount of 12 diterpene lactones standard reference and diluted to 50 μg/mL. Different parts of *A. paniculata* at different harvest ages that including sixteen batches of stem, sixteen batches of leaf, fifteen batches of root, four batches of flower, and five batches of fruit were divided, respectively (Table S2) [18].

### 2.3. Chromatographic Conditions and Mass Spectrometry Parameters

The *A. paniculata* samples were analyzed using an Ultimate 3000 Standard Dual System (Thermo Fisher Scientific, San Jose, CA, USA) equipped with a binary solvent pump, an autosampler, and a diode-array detector. The samples were separated on an ACQUITY HSS T3 C18 column (2.1 100 mm, 1.7 μm, Waters, Milford, MA, USA). The mobile phase consisted of water containing 0.1% (*v*/*v*) formic acid (A) and acetonitrile (B); the flow rate was set to 0.3 mL/min. During the sample elution process, samples were eluted with an initial flow phase equilibrium for 2 min, a linear gradient increase in mobile phase B from 2% to 20% for 3 min, a linear gradient increase in mobile phase B from 20% to 55% for 20 min, a linear gradient increase in mobile phase B from 55% to 70% for 5 min, a linear gradient increase in mobile phase B from 70% to 100% for 5 min, a liner gradient decrease in mobile phase B from 100% to 0% for 5 min. The column temperature was maintained at 35 °C, and the injection volume of the test solution was 2 μL. In addition, the detection wavelength was 205 nm.

The experiments were performed at a spray voltage of 4600 V, a capillary temperature of 350 °C, an in-source CID of 20 V, a collision energy of 30 V, and source heater temperature of 300 °C. Nitrogen was applied as sheath gas and auxiliary gas, and their flow rate was 40 and 10 arb units, respectively. Data were acquired in positive ionization mode.

The chromatographic conditions and mass spectrometry parameters for untargeted metabolomics analysis of *A. paniculata* at different harvest periods and plant parts were consistent with the methods used in previous studies [18]. The UHPLC-LTQ-Orbitrap Velos Pro hybrid mass spectrometer (Thermo Fisher Scientific, San Jose, CA, USA) was employed for data acquisition, using different methods based on the compound types. Firstly, the collision-induced dissociation (CID) mode was selected to acquire the first-level mass spectrometry data for compounds with molecular weights ranging from 100 to 1200 Da, recorded as Event I. Event II recorded the fragmentation ions of the top three ions in Event I, while Event III captured the fragment ion information of the top three ions in Event II. Specifically, the data acquisition method for diterpene lactones in *A. paniculata* was consistent with our previous publication [18]. For flavonoid constituents, a polygonal mass defect filtering method was developed based on a literature survey of 66 compounds to screen potential flavonoid constituents in *A. paniculata*, and their multi-stage mass spectrometry information was acquired (Figure S2). As for caffeoylquinic acid constituents, a polygonal mass defect filtering method was devised based on a literature survey of six compounds to screen potential caffeoylquinic acid constituents in *A. paniculata* (Figure S3), and their multi-stage mass spectrometry information was obtained.

### 2.4. Chemical Characterization

A database was established by collecting information on 201 reported compounds of *A. paniculata*, including compound names, structural formulas, molecular formulas, and CAS numbers, from databases such as ScienceFinder*^n^* (http://scifinder-n.cas.org/, accessed on 24 May 2023), PubMed (https://pubmed.ncbi.nlm.nih.gov/, accessed on 24 May 2023), Reaxys (https://www.reaxys.com/, accessed on 24 May 2023), and CNKI (https://c61.oversea.cnki.net/, accessed on 24 May 2023). The created compound database was imported into Compound Discoverer 3.3. Based on the characteristics of the constituents in *A. paniculata*, the following adduct ions were set in ESI^+^ mode: [M + H]^+^, [2M + H]^+^, [M + Na]^+^, [2M + Na]^+^, [M + H − H_2_O]^+^, [M + H − 2H_2_O]^+^, and [M + H − 3H_2_O]^+^, with a mass error tolerance of 5 ppm, and potential known constituents in *A. paniculata* were searched. Finally, the mass spectra fragments of characterized compounds were compared with those of reference standards and literature data to further confirm the chemical constituents in *A. paniculata*.

### 2.5. Data Analysis

The raw data of *A. paniculata* collected from different growth stages and plant parts were imported into Progenesis QI 2.1 software for baseline correction, smoothing, peak extraction, and deconvolution. The processing parameters were set as follows: file type was selected as profile format, adduct ions were set as [M + H]^+^, [2M + H]^+^, [M + Na]^+^, [2M + Na]^+^, [M + H − H_2_O]^+^, [M + H − 2H_2_O]^+^, and [M + H − 3H_2_O]^+^; the minimum peak intensity was set to 2000, and the maximum peak width was set to 0.2 min. A data matrix containing retention time, *m*/*z*, and peak intensity information was obtained. Signal noise ions that did not meet the 30% and 80% rule and had high responses in the blank solvent were removed from the metabolomics data processing, resulting in the dataset used for metabolomics analysis. Multivariate statistical analysis and clustering analysis of the metabolomics data were performed using SIMCA-P 14.1 software (Umetrics, Umea, Sweden) and HemI software (CUCKOO Workgroup, Wuhan, China). Metabolites with VIP (Variable Importance in Projection) values greater than 3 were further subjected to *t*-tests to identify potential quality markers more accurately. The quantitative analysis results were plotted using Origin 2019b software (OriginLab, Northampton, MA, USA).

## 3. Results and Discussion

### 3.1. Optimization of Chromatographic Conditions and Mass Spectrometry Parameters

**Figure 2:**
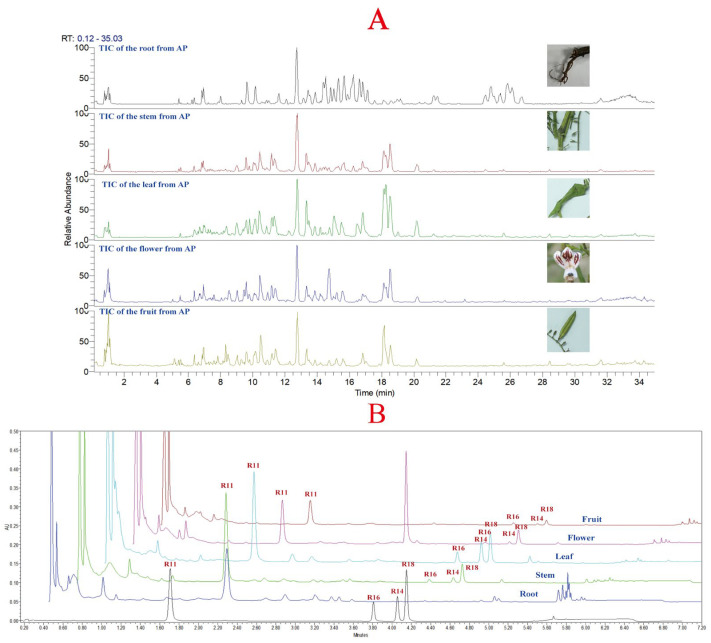
(**A**): Total ion chromatogram of different parts of *A. paniculata*; (**B**): chromatograms of different parts of *A. paniculata* (R11: andrographolide; R16: neoandrographolide; R14: 14-deoxyandrographolide; R18: dehydroandrographolide).

Due to the complexity and trace amounts of components in natural products, the chromatographic gradient directly affects the quality and quantity of compound data collected by mass spectrometry [19,20]. Therefore, it is necessary to optimize the chromatographic conditions before data acquisition. Similarly, the mass spectrometry parameters also affect the fragmentation pattern of compounds and thus influence their resolution [21]. In this study, the chromatographic conditions and mass spectrometry parameters used in metabolomics data acquisition were optimized, and the quantification of the four major diterpene lactones was performed using a method established in our laboratory for *A. paniculata* quality standard [18]. The total ion chromatogram of different plant parts of *A. paniculata* is shown in Figure 2A, and the chromatograms of different plant parts are shown in Figure 2B. In Figure 2A, it can be observed that there are significant differences in the constituents between the root of *A. paniculata* and other plant parts, while the stem, leaf, flower, and fruit show more similar profiles. The leaf of *A. paniculata* showed a higher concentration of constituents compared to the stem and leaf. Figure 2B shows that the four major diterpene lactones were not detected in the root of *A. paniculata*, while andrographolide, neoandrographolide, dehydroandrographolide, and 14-deoxyandrographolide were detected in the stem, leaf, flower, and fruit, with relatively higher levels in the leaf of *A. paniculata*.

### 3.2. Identification of Chemical Constituents in Different Growth Stages and Plant Parts of A. paniculata

#### 3.2.1. Analysis of Diterpene Lactones in *A. paniculata*

Diterpene lactones are the most abundant active components in *A. paniculata*, and their identification is of great significance [22]. However, the characterization of diterpene lactones in *A. paniculata* has been extensively reported in previous publications [18]. In this study, our focus was on the distribution of diterpene lactones in different plant parts of *A. paniculata*.

#### 3.2.2. Analysis of Flavonoids in *A. paniculata*

Flavonoids belong to the natural polyphenolic compounds and are widely distributed in common foods such as vegetables, fruits, and tea [23]. Existing studies have shown that flavonoids possess various biological activities, including antioxidant, anti-inflammatory, antibacterial, and antiviral effects [24]. However, it has been reported that flavonoids account for 46.23% of the reported constituents in *A. paniculata*, including flavones, flavonols, and dihydroflavones [2]. Therefore, it is necessary to systematically characterize the flavonoid constituents.

**Figure 3:**
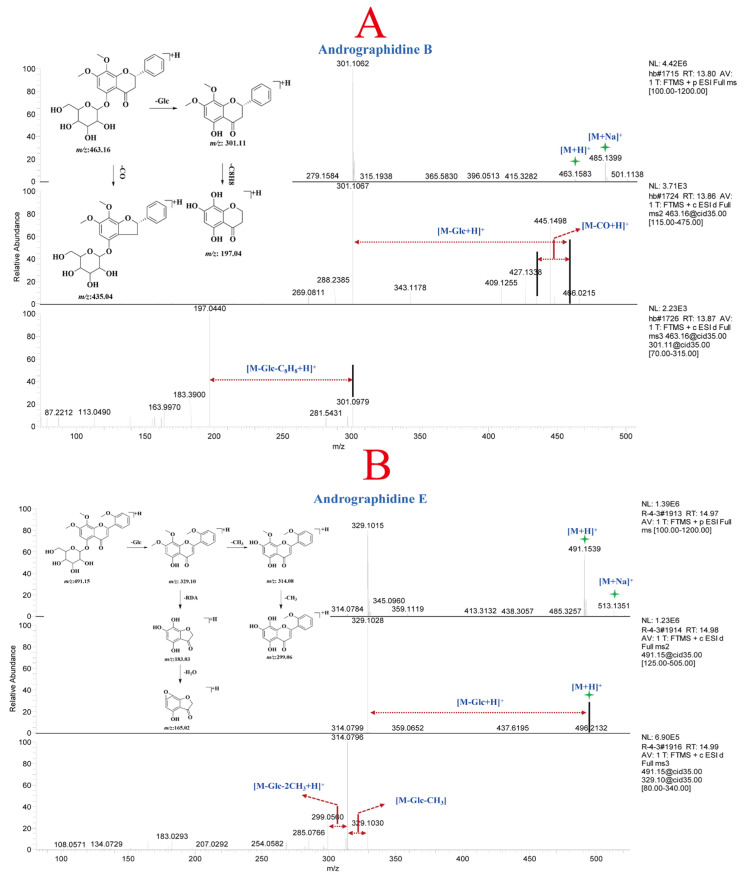
MS^n^ spectra and potential fragmentation patterns of andrographidine B (**A**) and andrographidine E (**B**).

Compounds **26** and **28** (Table S3) were positional isomers, both exhibiting molecular ion peaks at *m*/*z* 463.16 Da [M + H]^+^ and [M + Na]^+^ at *m*/*z* 485.14 Da in the protonated molecular ions. Furthermore, fragment ion peaks at *m*/*z* 301.11 Da, and 197.04 Da were detected in the MS^n^ analysis. The fragment ion at *m*/*z* 301.11 Da was the aglycone formed by the loss of one molecule of Glc. The fragment ion at *m*/*z* 197.04 Da represented the loss of one molecule of Glc and two molecules of CH_2_ from compounds **26** and **28**. By comparing the retention time, *m*/*z*, and MS^n^ fragment patterns with reference standards, compounds **26** and **28** were identified as andrographidine A and andrographidine B, respectively, belonging to the dihydroflavone class. The mass spectrum and potential fragmentation pattern of compound **28** are shown in Figure 3A. Compound **36** exhibited a molecular ion peak at *m*/*z* 491.15 Da [M + H]^+^ and an adduct ion peak [M + Na]^+^ at *m*/*z* 513.14 Da in the MS. Fragment ion peaks at *m*/*z* 329.10, 314.08, 299.06, 183.03, and 165.02 Da were detected in the MS^n^ analysis. The fragment ion at *m*/*z* 329.10 Da mainly resulted from the loss of one molecule of Glc from the precursor ion, while the fragment ions at *m*/*z* 314.08 Da and 299.06 Da corresponded to the consecutive loss of two CH_3_ groups from *m*/*z* 329.10 Da. The fragment ions at *m*/*z* 183.03 Da and 165.02 Da were generated by the RDA fragmentation of the parent nucleus of compound **36**. After comparing the retention time, *m*/*z*, and mass spectral fragments with reference standards, compound **36** was identified as andrographidine E. The potential fragmentation pattern of andrographidine E is shown in Figure 3B. Finally, a total of 72 flavonoid constituents (Table S3, 1–72) were characterized in *A. paniculata* based on the mass spectral fragments of reference compounds and literature data.

#### 3.2.3. Analysis of Organic Acids in *A. paniculata*

Organic acids are common constituents in plant species, and pharmacological studies have shown that these compounds possess various biological activities such as anti-inflammatory, antioxidant, and neuroprotective effects [25,26,27,28]. Thus, characterizing the organic acids in *A. paniculata* is of significance. In this study, based on the reported organic acids in *A. paniculata*, a polygonal mass defect window was established, and 190 potential organic acids were screened from the leaves of *A. paniculata*. Subsequently, the potential organic acids in *A. paniculata* were collected using a precursor ion list approach.

**Figure 4:**
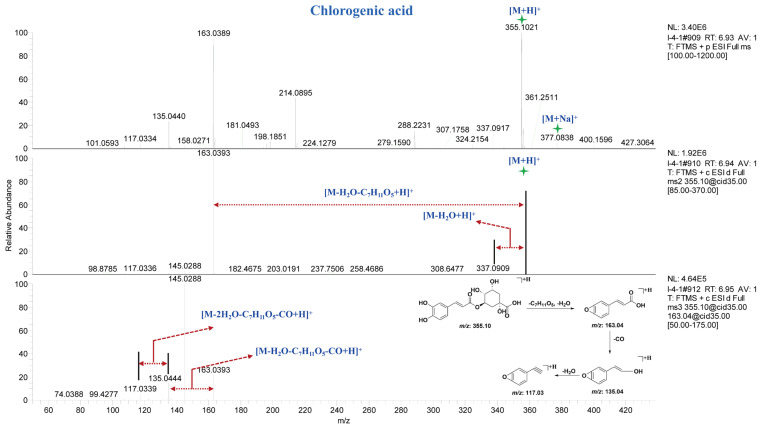
MS^n^ spectra and potential fragmentation patterns of chlorogenic acid.

Compound **76** exhibited a [M + H]^+^ ion peak at *m*/*z* 355.1014 Da and a [M + Na]^+^ ion peak at *m*/*z* 377.083 Da in the MS spectrum. In the MS^2^ spectrum, fragment ion peaks at *m*/*z* 337.09 Da and *m*/*z* 163.04 Da were observed, corresponding to the loss of one molecule of H_2_O and C_7_H_11_O_5_ from the precursor ion, respectively. In the MS^3^ spectrum, fragment ion peaks at *m*/*z* 135.04 Da and *m*/*z* 117.03 Da were detected, which originated from the *m*/*z* 163.04 Da fragment with higher spectral intensity in the MS^2^ spectrum. Through comparison with reference standards in terms of retention time and mass spectral fragmentation, compound **76** was identified as chlorogenic acid. The MS, MS^2^ and MS^3^ spectra and potential fragmentation patterns of compound **76** are shown in Figure 4. Finally, through comparison with reference standards and literature data, ten caffeoylquinic acid compounds (Table S3, 74–82) were characterized from different parts of *A. paniculata*.

#### 3.2.4. Analysis of Other Components in *A. paniculata*

Previous reports have indicated that besides diterpene lactones, flavonoids, and organic acids, *A. paniculata* also contains other types of compounds such as triterpenoids and adenosine [3]. To systematically characterize other components in *A. paniculata*, the raw data obtained were imported into Compound Discoverer 3.3 software for local database identification and network database searching.

**Figure 5:**
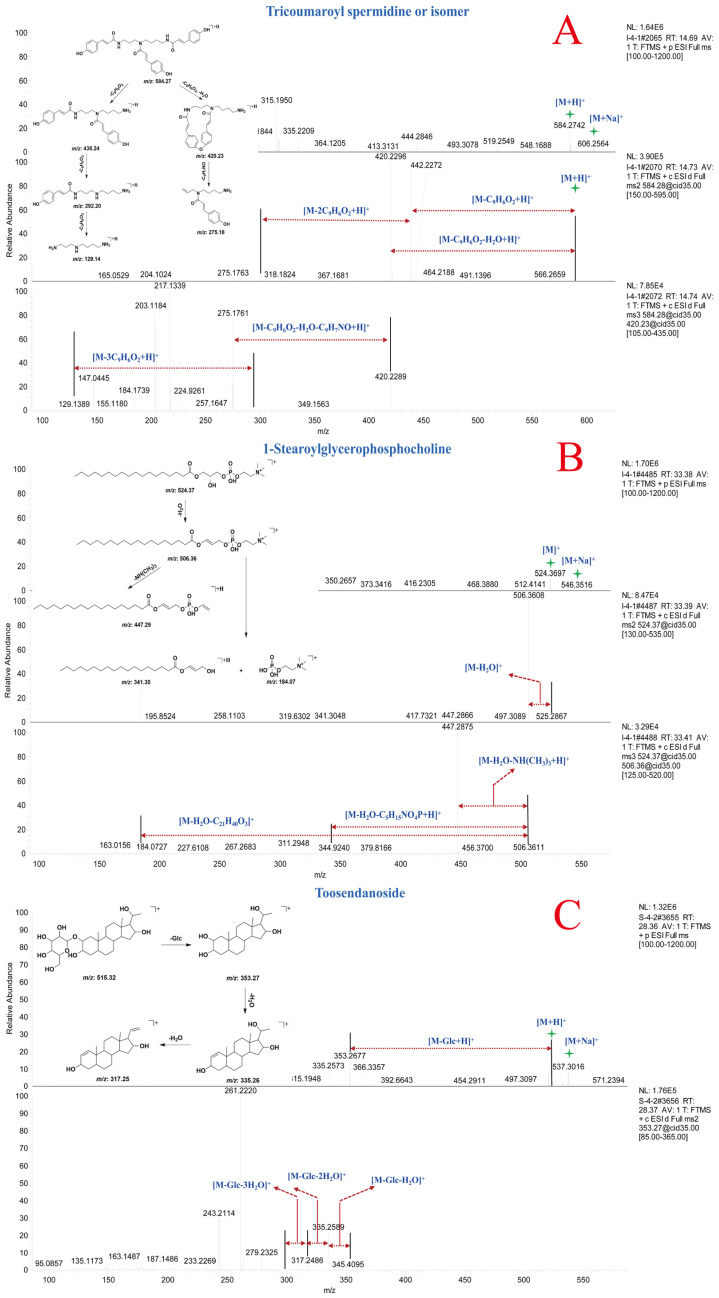
MS^n^ spectra and potential fragmentation patterns of tricoumaroyl spermidine or isomer (**A**), 1-stearoylglycerophosphocholine (**B**), and toosendanoside (**C**).

Compound **83** and **84** exhibited [M + H]^+^ ion peaks at *m*/*z* 584.27 Da and [M + Na]^+^ ion peaks at *m*/*z* 606.25 Da, indicating that compounds **83** and **84** are a pair of isomeric compounds. In the MS^n^ spectra, fragment ion peaks at *m*/*z* 438.24, 420.23, 292.20, 275.18, and 129.14 Da were detected. The fragment ions at *m*/*z* 438.24, 292.20, and 129.14 Da corresponded to the consecutive loss of three molecules of coumaroyl from the precursor ion. The fragment ions at *m*/*z* 420.23 Da and *m*/*z* 275.18 Da resulted from the loss of one molecule of coumaroyl, H_2_O, and C_9_H_7_NO from the precursor ion, respectively. Through comparison with online mass spectral databases, compounds **83** and **84** were identified as tricoumaroyl spermidine or an isomer. The MS^n^ spectra and potential fragmentation patterns of compounds **83** and **84** are shown in Figure 5A [29]. The fragment ions at *m*/*z* 438.24, 420.23, 292.20, 275.18, and 129.14 Da could serve as diagnostic ions for spermidine derivatives in natural products.

Compound **93** was identified as 1-stearoylglycerophosphocholine by comparing its MS^n^ data with the spectral information in the online spectral databases and preliminary research [30]. In the MS spectrum, compound **93** displayed [M + H]^+^ ion peak at *m*/*z* 524.37 Da and [M + Na]^+^ ion peak at *m*/*z* 546.35 Da. In the MS^2^ and MS^3^ spectra, fragment ions at *m*/*z* 506.36, 447.29, 341.30, and 184.07 Da were detected. The fragment ion at *m*/*z* 506.36 Da resulted from the loss of one molecule of H_2_O from the precursor ion. Fragment ions at *m*/*z* 447.29, 341.30, and 184.07 Da were derived from the fragment ion at *m*/*z* 506.36 Da through subsequent losses of NH(CH_3_)_3_, C_5_H_15_NO_4_P, and C_21_H_40_O_3_, respectively. The MS^n^ spectrum and potential fragmentation patterns of compound **93** are shown in Figure 5B. Based on the characteristic fragment ions in the mass spectrum, compounds **86**–**93** were identified as phosphatidylcholines.

Compound **103** exhibited [M + H]^+^ ion peak at *m*/*z* 515.32 Da and [M + Na]^+^ ion peak at *m*/*z* 537.30 Da in MS spectrum. In the MS^n^ spectrum, fragment ion peaks at *m*/*z* 353.27, 335.26, 317.25, and 259.08 Da were detected, corresponding to consecutive loss of one molecule of Glc and two molecules of H_2_O from the precursor ion at *m*/*z* 515.32 Da. Comparison with literature mass spectral data confirmed that compound **103** was toosendanoside [31]. The MS^n^ spectrum and potential fragmentation patterns of toosendanoside are shown in Figure 5C. Compared to compound **103**, compound **100** exhibited an *m*/*z* of 677.37 Da, indicating the presence of an additional molecule of Glc, and the glycone portion showed consistent fragmentation patterns. Based on the characteristic fragment ions in the mass spectrum, compounds **94**–**106** were identified as triterpenoids.

Consequently, through comparison with reference standards, the literature, and online mass spectral databases, a total of 126 compounds were characterized from *A. paniculata*, excluding diterpene lactones. The characterized compounds included 72 flavonoids, ten caffeoylquinic acid derivatives, three spermidine derivatives, eight phosphatidylcholines, 13 triterpenoids, and 20 other components. In the identified components, we identified 67, 42, 29, 30, and 20 flavonoids in the roots, stems, leaves, flowers, and fruits of *A. paniculata*, respectively. Additionally, we detected 9, 9, 9, 7, and 7 sphenylpropanoid in the roots, stems, leaves, flowers, and fruits, respectively. Spermidine derivatives and phosphatidylcholines were found to be present in all parts of *A. paniculata*. Furthermore, we identified 12, 12, 7, 5, and 5 triterpenoids in the roots, stems, leaves, flowers, and fruits, respectively. In addition, 15, 17, 16, 15, and 16 of other types of compounds were identified in the roots, stems, leaves, flowers, and fruits of *A. paniculata*, respectively.

### 3.3. Metabolite Analysis of A. paniculata at Different Growth Stages and Plant Parts

Previous studies have shown significant variations in the content of major diterpenoid lactones in *A. paniculata* leaves from different manufacturers and batches, which could be attributed to variations in harvesting time and inconsistent leaf-to-stem ratios [32,33]. To explore the differential metabolites in *A. paniculata* at different growth stages and plant parts, a non-targeted metabolomics approach was employed in this study. Raw data from 56 samples of *A. paniculata* roots, stems, leaves, and seven batches of quality control (QC) samples were imported into Progenesis QI 2.1 software for peak alignment and extraction, facilitating subsequent targeted. Finally, the exported data matrix is used for non-targeted metabolomics analyses.

#### 3.3.1. Targeted Analysis of Major Compounds in *A. paniculata*

To investigate the distribution of diterpenoid lactones and flavonoids in different parts of *A. paniculata*, a polygonal mass defect filter was applied to screen potential diterpenoid lactones and flavonoids. In the roots of *A. paniculata*, 4342 ions were detected, and after the targeted metabolite screening using the polygonal mass defect filter, 556 potential diterpenoid lactones and 778 potential flavonoids were identified. Similarly, 4809 ions were detected in the stems of *A. paniculata*, and after the screening process, 868 potential diterpenoid lactones and 642 potential flavonoids were identified. In the leaves of *A. paniculata*, 4717 ions were detected, and the targeted analysis revealed 1550 potential diterpenoid lactones and 426 potential flavonoids. Due to the limited sample numbers of flowers and fruits, their data were combined for analysis. By applying the polygonal mass defect filter, 4385 ions were detected in the flowers and fruits of *A. paniculata*, including 886 potential diterpenoid lactones and 493 potential flavonoids. These results indicate that diterpenoid lactones were predominantly present in *A. paniculata* leaves, while flavonoids were relatively less diverse in this plant part. The roots exhibited a higher abundance of flavonoids compared to diterpenoid lactones, whereas the stems displayed the highest diversity of compounds, including a wide range of diterpenoid lactones and flavonoids. Flowers and fruits mainly contained diterpenoid lactones, with fewer flavonoid compounds.

#### 3.3.2. Non-Targeted Metabolomics Analysis of Chemical Constituents in *A. paniculata*

**Figure 6:**
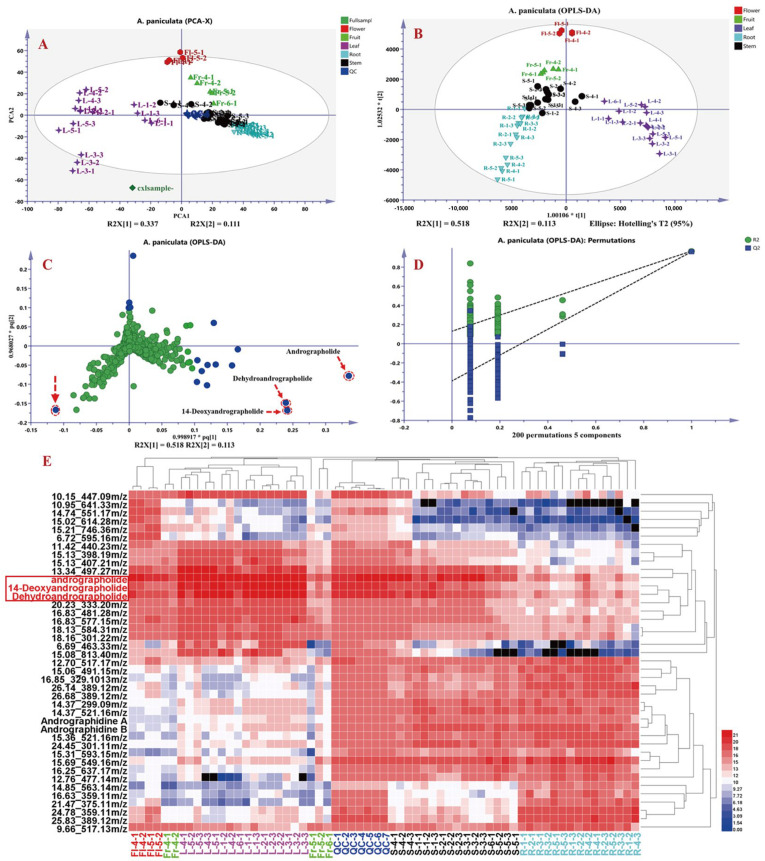
Principal component analysis (PCA) and cluster analysis results of different parts in *A. paniculata*. PCA plot (**A**), orthogonal partial least squares discriminant analysis (OPLS-DA) plot (**B**), OPLS-DA loading plot (**C**), OPLS-DA model validation results, *: *p* < 0.05 (**D**), and heatmap of differential metabolites (**E**).

To further explore differential metabolites in *A. paniculata* from different origins and medicinal plant parts, the raw data were processed using Progenesis QI 2.1 software, resulting in a data matrix containing 6192 variables, including retention time, *m*/*z*, and ion intensities. Subsequently, based on the “30% rule” and “80% rule” in metabolomics data analysis, 1969 data points that did not meet the criteria were excluded using Excel formulas, resulting in a final data matrix of 4223 variables. The processed data matrices (63 in total) were then imported into SIMCA-P 14.1 software for principal component analysis (PCA) and orthogonal partial least squares discriminant analysis (OPLS-DA). As shown in Figure 6, Figure 6A presents the PCA plot of the 63 datasets, with the seven QC samples tightly clustered, indicating good instrument precision and reproducibility for further analysis. Figure 6B shows the OPLS-DA results of different parts of *A. paniculata*, revealing clear differentiation among roots, stems, leaves, flowers, and fruits, indicating the presence of chemical composition variations across different plant parts. The loading plot in Figure 6C highlights andrographolide, dehydroandrographolide, and 14-deoxyandrographolide as the key differential compounds influencing the classification of *A. paniculata* plant parts. The OPLS-DA model exhibited good fitting (R2Y(cum) = 0.951) and predictive ability (Q2(cum) = 0.933), as validated through 200 permutations [i.e., R2 = (0.0, 0.157), Q2 = (0.0, −0.444)], indicating robustness and reliability. Subsequently, variables with VIP (Variable Importance in Projection) values greater than three were selected and further analyzed using *t*-tests in MetaboAnalyst 5.0 online platform. Variables with VIP values greater than 3 and *p*-values less than 0.05 were considered as differential compounds influencing the classification of *A. paniculata* plant parts (Table S4). A total of 41 compounds were selected as differentiating factors. To visually illustrate the impact of these 41 variables on the classification of *A. paniculata* plant parts, the mass spectrometry responses of these compounds in different plant parts were imported into HemI 2.0 software for cluster analysis. The cluster analysis included seven QC samples, 15 root samples, 16 stem and leaf samples, five fruit samples, and four flower samples, and employed the square Euclidean distance method. The results in Figure 6E demonstrate the classification of the 63 datasets into three main clusters, with distinct separation of roots from stems and leaves. Leaves and stems also exhibited good differentiation, while flowers and early-stage fruits showed similar profiles to leaves, with higher levels of diterpenoid lactones and lower levels of flavonoids. QC samples and later-stage fruits were more similar to stems, with higher levels of flavonoids compared to *A. paniculata* leaves. The identified differential compounds included andrographolide, andropanoside, dehydroandrographolide, 14-deoxyandrographolide, andrographidine A, and andrographidine B, which could be served as characteristic markers for distinguishing different parts of *A. paniculata*. According to the heat map analysis, it can be seen that the content of the three main differential components in the third and fourth week of *A. paniculata* are more significant than those in other stages, and the proportion of roots and stems and yield are comprehensively analyzed, and the yield in the third week is less, so we believe that the collection time of the fourth week after planting is the best harvest time for *A. paniculata*.

## 4. Conclusions

In this study, a systematic characterization of chemical components in *A. paniculata* was performed, followed by the analysis of differential metabolites in different parts of the plant at various harvest periods using a non-targeted metabolomics approach. A total of 225 components were identified, including 99 diterpene lactones 18, 72 flavonoids, 10 caffeoylquinic acids, 3 spermidine derivatives, 8 phosphatidylcholines, 13 triterpenoids, and 20 other types of components. Notably, several component types, such as spermidine derivatives and phosphatidylcholines, were reported for the first time in *A. paniculata*. In addition to the four major diterpene lactones, 37 other components, including andrographidine A and andrographidine B, could serve as potential quality markers for the identification of raw materials in *A. paniculata* products. In addition to this, we also consider the fourth week after planting to be the best time to harvest *A. paniculata*. Our study provides important insights into the chemical composition and differential metabolites in different parts of *A. paniculata* and offers references for the selection of quality markers in *A. paniculata* herbal materials and related products.

## Data Availability

The original contributions presented in this study are included in the article/Supplementary Material. Further inquiries can be directed to the corresponding authors.

## Supplementary Materials

The following supporting information can be downloaded at: https://www.mdpi.com/article/10.3390/molecules30071490/s1, Figure S1: Determine results of four diterpene lactones in Chuanxinlian tablets from different manufacturers. Figure S2: Polygonal mass deficit map of flavonoids in *A. paniculata*. Figure S3: Polygonal mass deficit map of caffeoyl quinic acid components in *A. paniculata*. Table S1: Sample information of Chuanxinlian tablets from different manufacturers. Table S2: Collect information of samples from different parts of *A. paniculata* at different growth stages. Table S3: Detailed information about the characterized components in *A. paniculata*. Table S4: Components with VIP > 3 between different harvest periods and different plant parts of *A. paniculata* based on the OPLS-DA.
