# Supplementary material for: Metabolomic Profiles and Differential Constituents of Andrographis paniculata (Burm. f.) in Different Growth Stages and Parts
Source: Molecules. 2025 Mar 27;30(7):1490. doi: 10.3390/molecules30071490 (PMC11990188; doi:10.3390/molecules30071490)
Supplement: Supplementary file 1 [file molecules-30-01490-s001.zip › Supplementary data.pdf]

Metabolomic profiles and differential constituents of *Andrographis paniculata* (Burm. f.) in different growth stages and parts

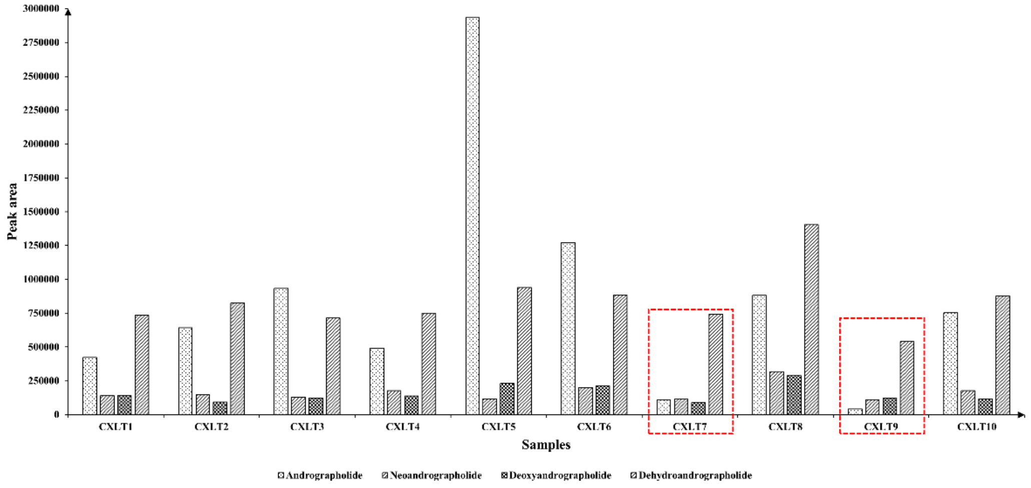

Figure S1. Determine results of four diterpene lactones in Chuanxinlian tablets from different manufacturers.

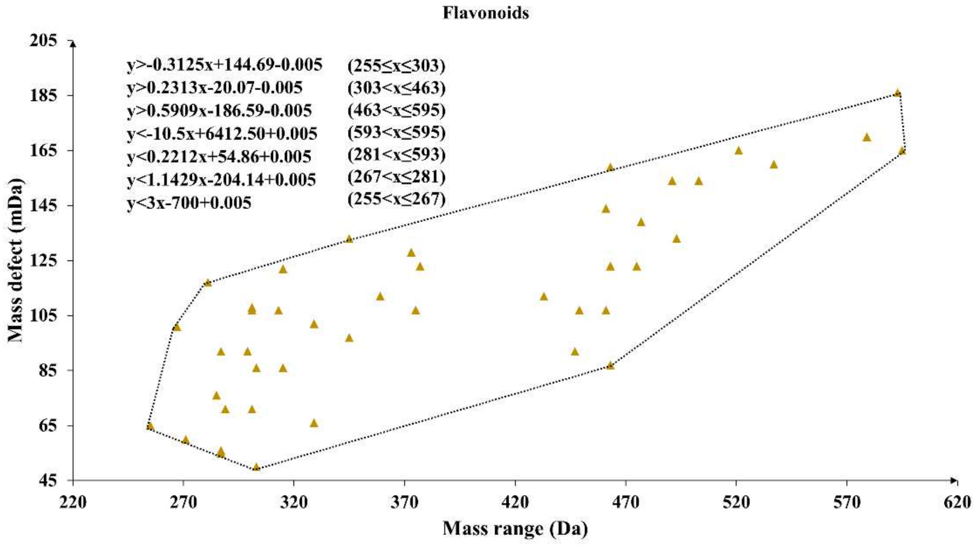

Figure S2. Polygonal mass deficit map of flavonoids in *A. paniculata*.

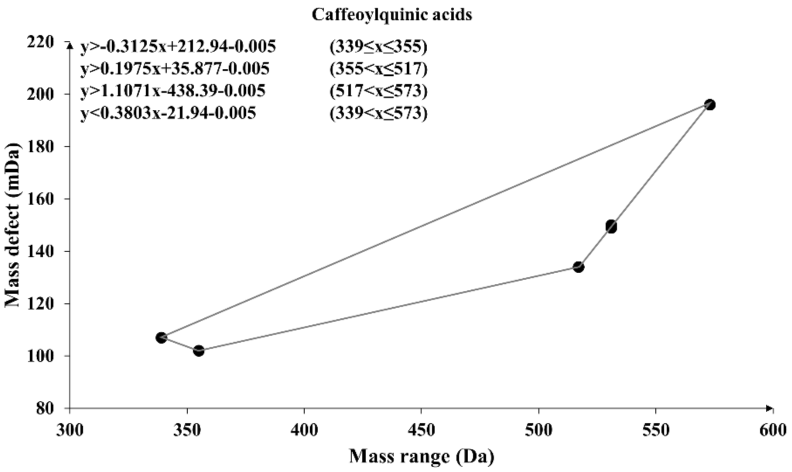

Figure S3. Polygonal mass deficit map of caffeoyl quinic acid components in *A. paniculata*.

Table S1 Sample information of Chuanxinlian tablets from different manufacturers

NO.	Manufacturer	Lot number
CXLT1	Sunflower Pharmaceutical Group Hubei Wudang Co.,Ltd	200501
CXLT2	Guangxi Fanglue Pharmaceutical Group Co., Ltd	2005021
CXLT3	Jilin Wantong Pharmaceutical Group Meihe Pharmaceutical Co., Ltd	20191101
CXLT4	Jilin Changheng Pharmaceutical Co., Ltd	20200306/081
CXLT5	Shanxi Canon Dahua Yu Pharmaceutical Co., Ltd	180803
CXLT6	Guangxi Jimin Pharmaceutical Co., Ltd	2004002
CXLT7	Hutchison Whampoa Guangzhou Baiyunshan Chinese Medicine Co., Ltd.	B20A002
CXLT8	Guangxi Jiajin Pharmaceutical Co.,Ltd.	2007001
CXLT9	Tai Chi Group Co., Ltd.	2019001
CXLT10	Shijiazhuang Yiling Pharmaceutical Co., Ltd.	A2001001

Table S2 Collect information of samples from different parts of *A. paniculata* at different growth stages

No.	Collection time	Number of the root samples	Number of the stem samples	Number of the leaf samples	Number of the flower samples	Number of the fruit samples
First time	June 12, 2020	3	3	3	-	-
Second time	July 16, 2020	3	3	3	-	-
Third time	August 1, 2020	3	3	3	-	-
Fourth time	August 21, 2020	3	3	3	2	2
Fifth time	September 10, 2020	3	3	3	2	2
Sixth time	October 5, 2020	-	1	1	-	1

"-" Means that no samples were collected at that time.

Table S3 Detailed information about the characterized components in *A. paniculata*.

NO.	Type	tR(min)	Identification	Formula	RDB	Error(ppm)	[M+H]+	[M+Na]+	Major fragment ions	Source
1	Flavonoids	6.72	6-C-β-D-Glucopyranosyl-8-C-β-D-galactopyranoside-apigenin	C27H30O15	12.5	-1.17	595.164	617.1464	449.11, 287.06, 219.07, 179.03, 161.02	Fr
2	Flavonoids	7.12	5,4'-Dihydroxy-7-O-β-D-pyranglycuronate ethyl ester	C22H18O12	13.5	-1.25	475.0858		299.06, 271.06, 199.02, 181.01	R, L, S, Fl, Fr
3	Flavonoids	7.81	Isoscutellarein-8-O-β-D-glucuronide	C21H20O11	11.5	-0.69	449.1071	471.0889	287.06, 237.04, 219.03, 203.03, 161.02	R
4	Flavonoids	7.83	Moslosooflavone+Glc	C23H24O10	11.5	-3.74	461.1425	483.1238	299.09, 284.07, 255.07, 238.06	R, Fr
5	Flavonoids	7.97	Violanthin	C27H30O14	12.5	-1.58	579.1693	601.1506	561.16, 543.15, 525.14, 495.13, 459.13, 441.12, 393.10, 363.09, 309.08, 291.06	R, L, S, Fr
6	Flavonoids	8.51	Keracyanin	C27H30O15	12.5	-2.09	595.1645		449.11, 433.11, 329.07, 287.06	R, S, L, Fl, Fr
7	Flavonoids	8.55	Rutin	C27 H30O16	12.5	-1.52	611.1598	633.1409	593.32, 465.10, 303.05, 285.04, 257.05, 229.05, 165.02	S, L, Fl, Fr
8	Flavonoids	8.9	Luteolin-7-O-β-D-glucuronide	C21H18O12	12.5	-0.16	463.0		287.06, 263.06,	R, L, S,

		6					869		179.03, 161.02	Fl, Fr
9	Flavonoids	9.50	8-[(2S,3R,4S,5S,6R)-4,5-Dihydroxy-6-(hydroxymethyl)-3-{[(2S,3R,4R,5R,6S)-3,4,5-trihydroxy-6-methyloxan-2-yl]oxy}oxan-2-yl]-5,7-dihydroxy-2-(4-hydroxyphenyl)-4H-chromen-4-one	C27 H30O14	12.5	-2.30	579.1695	601.1501	433.11, 381.10, 313.06, 271.06	L, S, Fl, Fr
10	Flavonoids	9.63	Tricin 5-O-β-D-glucoside	C23 H24O12	11.5	-0.80	493.1328	515.1148	331.0812, 316.0602	R, S
11	Flavonoids	9.71	Unknown	C28H32O15	12.5	-0.37	609.1811	631.1628	463.13, 301.07	S, L, Fl, Fr
12	Flavonoids	10.04	Unknown	C33H38O22	14.5	0.08	787.1928	809.1731	625.14, 463.09, 287.06	R, L, S, Fl, Fr
13	Flavonoids	10.18	Apigenin-7-O-glucuronide	C21H18O11	12.5	-3.78	447.0905	499.1212	271.06, 225.06, 153.02	R, L, S, Fl, Fr
14	Flavonoids	10.96	Unknown	C24H26O12	11.5	-1.63	507.1481	529.1298	345.10, 330.07, 315.05, 301.07, 284.07, 197.04, 183.03, 165.02	R, L, S
15	Flavonoids	11.25	Unknown	C23H26O8	10.5	-3.32	431.1702		389.12, 374.10, 359.11, 344.09, 298.08, 197.04, 183.03, 165.02	R, S
16	Flavonoids	11.42	Unknown	C26H24O11	14.5	-3.52	513.1378	535.1442	333.10, 319.08, 273.08, 177.05, 163.04	R, L, S, Fl, Fr
17	Flavonoids	11.49	Unknown	C25H24O14	13.5	-1.26	549.1226	571.1051	531.15, 367.12, 301.11, 259.10, 197.04	R
18	Flavonoids	11.6	5,4'-Dihydroxy-7,8,2,3'-tetramethoxyflavone+Glc	C25H28O13	11.5	-2.36	537.1	559.1	375.11, 342.07,	R, L, S,

		3					59	401	327.05, 197.05, 183.03, 165.02	Fl, Fr
19	Flavonoids	11.91	Unknown	C33H54O10	6.5	-2.26	611.3767		449.33, 305.21, 287.20, 251.18, 241.20	Fl
20	Flavonoids	12.02	Isomer of andrographidine E	C24H26O11	11.5	-1.53	491.1533	513.1368	329.10, 314.08, 299.06, 285.08	R
21	Flavonoids	12.08	Andrographidine E+CO2	C25H26O13	12.5	-1.75	535.1437	557.1255	331.08, 316.06, 298.05,287.06, 197.04	R
22	Flavonoids	12.72	Skullcapflavone I 2’-O-β-D-glucopyranoside	C23H24O11	11.5	-3.10	477.1377	499.1194	315.09, 300.06, 282.05, 271.06, 254.06	R, L, S, Fl, Fr
23	Flavonoids	12.77	Unknown	C25H24O12	12.5	-3.91	505.1321	527.1141	301.07, 273.07, 233.04, 203.03, 167.03	R, L, S, Fr
24	Flavonoids	13.14	Unknown	C26H28O14	12.5	-2.22	565.1538	587.1353	547.14, 317.10, 299.09, 275.09, 197.04, 183.03, 165.02	S, R
25	Flavonoids	13.35	Unknown	C28H30O16	13.5	-0.99	623.1597	645.1411	405.12, 390.10, 375.07, 357.06, 329.07, 197.04, 183.03, 165.02	R
26(R5)	Flavonoids	13.53	**Andrographidine A**	C23H26O10	10.5	-3.20	463.1584	485.1402	301.11, 197.04	S, R
27	Flavonoids	13.	Unknown	C26H28O14	12.5	-2.43	565.1	587.1	547.15, 317.10,	S, R

		80					539	359	275.09, 223.06, 197.04	
28(R6)	Flavonoids	13.89	**Andrographidine B**	C23H26O10	10.5	-3.07	463.1578	485.1397	301.11, 197.04	S, R
29	Flavonoids	14.04	Unknown	C29H32O17	13.5	-1.06	653.1702	675.1515	635.16, 405.12, 390.09, 375.07, 357.06, 197.04, 183.03, 165.02	S, R
30	Flavonoids	14.38	495.2571-H2O-O	C23H24O10	11.5	4.05	461.1429	483.1245	299.09, 284.07, 255.07	R, L, S, Fl, Fr
31	Flavonoids	14.40	Andrographidine E+CH2O	C25H28O12	11.5	-2.21	521.1642	543.1455	359.11, 344.09, 329.07, 298.08	R, Fl, Fr
32	Flavonoids	14.43	Andrographidine G	C23H24O11	11.5	-1.26	477.1379	499.0992	315.09, 300.06, 297.09, 282.05, 271.06, 254.06, 197.04	R
33	Flavonoids	14.51	Unknown	C26H26O14	13.5	-1.10	563.1384	585.1202	545.17, 359.11, 344.09, 329.07, 298.08, 197.04, 183.03, 165.02	R, L, S
34	Flavonoids	14.57	Andrographidine C	C23H26O10	10.5	-1.50	463.1592	485.1404	301.11, 259.10, 197.04	S, R
35	Flavonoids	14.84	Andrographidine E+2CH2O	C26H30O13	11.5	-1.90	551.1749	573.1567	389.12, 374.10, 359.11, 344.09, 298.08, 197.04, 183.03, 165.02	S, R

36(R10)	Flavonoids	15.03	**Andrographidine E**	C24H26O11	11.5	-4.66	491.1525	513.1336	329.1022, 314.0793, 299.0557, 183.03, 165.02	R, L, S, Fl, Fr
37	Flavonoids	15.19	Unknown	C26H28O13	12.5	-1.82	549.1584	571.1403	531.15, 513.14, 409.13, 343.12, 301.11, 239.06, 197.04	R, L, S, Fl, Fr
38	Flavonoids	15.37	5-Hydroxy-7,8,2',5'-tetramethoxyflavone 5-O-glucoside+Glc	C25H28O12	11.5	-2.37	521.1641	543.1459	359.11, 344.08, 09, 329.07, 298.08, 197.04, 183.03, 165.02	R, Fl, Fr
39	Flavonoids	15.79	Puerarin xyloside	C26H28O13	12.5	-2.41	549.1587	571.1396	531.15, 513.14, 409.13, 367.12, 343.12, 301.11, 239.04, 197.04	R, L, S, Fl, Fr
40	Flavonoids	15.97	Unknown	C26H26O14	13.5	-1.29	563.1382	585.1197	545.13, 477.14, 315.19, 300.06, 282.05, 271.06, 254.06	R, L, S
41	Flavonoids	16.08	Unknown	C28H30O15	13.5	-0.67	607.1653	629.1458	589.15, 359.11, 344.09, 329.07, 298.08, 197.04, 183.03, 165.02	R
42	Flavonoids	16.21	Unknown	C26H26O13	13.5	-1.80	547.1428	569.1241	299.09, 284.07, 255.07, 207.03	R, L, S, Fl, Fr
43	Flavonoids	16.	Unknown	C29H32O16	13.5	-1.14	637.1	659.1	389.12, 374.10,	R

		24					752	565	359.08, 341.07, 205.05, 197.04, 183.03, 165.02	
44	Flavonoids	16.51	Unknown	C29H32O16	13.5	-1.79	637.1752	659.1567	619.17, 389.12, 374.10, 359.08, 328.10, 296.07, 257.08, 197.04, 183.03, 165.02	R, S, Fl
45	Flavonoids	16.90	C26H27O11+CO2	C27H28O14	13.5	-2.48	577.1537	599.1346	559.15, 371.11, 329.10, 314.08, 299.06, 285.08	S, R
46	Flavonoids	17.07	Unknown	C28H30O15	13.5	-1.77	607.1647	629.1451	589.15, 359.11, 344.09, 329.07, 298.08, 197.05, 183.03, 165.02	R, L, S, Fl, Fr
47	Flavonoids	17.56	Unknown	C25H28O11	11.5	-1.74	505.1687	527.1502	423.09, 323.09, 307.06, 237.04, 227.05	S, R, L
48	Flavonoids	17.88	Unknown	C27H30O13	12.5	-2.73	563.1744	585.1563	545.16, 359.11, 344.09, 329.07, 298.08, 197.05, 183.03, 165.02	R
49	Flavonoids	18.23	Unknown	C25H26O11	12.5	-1.16	503.1536	525.1353	299.09, 284.07, 255.07, 207.03	S, R, L
50	Flavonoids	18.35	2-(3,4-Dihydroxy-5-methoxyphenyl)-2,3-dihydro-7-methoxy-4H-1-benzopyran-4-one	C17H16O6	9.5	-1.37	317.1015	339.0845	299.20, 287.20, 275.09, 197.04,	R, S

									182.02	
51	Flavonoids	18.55	5,7,4'-Trimethoxyflavone	C18H16O5	10.5	-1.15	313.1067	335.0883	298.08, 283.06, 269.08, 197.04, 183.03, 165.02	R, S, Fl
52	Flavonoids	18.55	Unknown	C27H30O13	12.5	-2.73	563.1744	585.1563	545.17, 359.11, 344.09, 329.07, 298.08, 197.04, 183.03, 165.02	R
53	Flavonoids	18.79	Unknown	C26H26O11	13.5	-2.25	533.1639	555.1454	515.15, 329.10, 314.08, 299.06, 285.08	S, R, L
54	Flavonoids	18.85	Unknown	C20H20O7	10.5	-2.63	373.1271	395.1088	343.08, 325.10, 297.08, 281.08, 265.05, 223.06, 197.04	R, S, Fl, Fr
55	Flavonoids	18.95	Unknown	C33H54O8	6.5	-1.88	579.3873	601.368	417.34, 273.22, 255.21	R
56(R7)	Flavonoids	19.04	**4',5,7,8-Tetramethoxyflavone**	C19H18O6	10.5	-3.57	343.1164	365.0981	328.09, 313.07, 298.08, 285.07, 257.08	R, L, S, Fl, Fr
57	Flavonoids	20.34	Unknown	C27H30O11	12.5	-3.08	531.1741	553.1663	449.11, 323.09, 253.07, 219.03, 182.07	R
58	Flavonoids	20.69	2-(3,4-Dihydroxy-5-methoxyphenyl)-2,3-dihydro-7-methoxy-4H-1-benzopyran-4-one+CO	C18H16O7	10.5	-2.35	345.0961	367.0777	330.07, 315.05, 197.04, 169.01	R, S
59	Flavonoids	21.	Isomer of skullcapflavone I	C17H14O6	10.5	-1.98	315.0	337.0	300.06, 282.05,	R, S, Fl,

		23					857	67	271.06, 254.06, 197.04, 181.01	Fr
60(R9)	Flavonoids	21.30	**Skullcapflavone I**	C17H14O6	10.5	-4.81	315.0848	337.066	300.06, 282.05, 271.06, 254.06	R
61	Flavonoids	21.41	5,4'-Dihydroxy-7,8,2,3'-tetramethoxyflavone	C19H18O8	10.5	-2.49	375.1065	397.0879	360.08, 342.07, 327.05, 197.04, 165.02	R, L, S, Fl, Fr
62	Flavonoids	22.13	Unknown	C20H20O9	10.5	-1.01	405.1171	427.0986	375.08, 329.07, 209.08, 197.04, 183.03, 165.02	R, S
63	Flavonoids	22.74	Skullcapflavone I+CH2O	C18H16O7	10.5	-2.26	345.0961	367.0775	300.07, 312.06, 284.07, 266.06	R, S
64(R4)	Flavonoids	24.47	**5-Hydroxy-7,8-dimethoxyflavanone**	C17H16O5	9.5	-4.48	301.1057	323.087	286.08, 259.10, 197.04, 182.02, 164.01	R, L, S, Fl, Fr
65	Flavonoids	24.74	Isomer of 5-hydroxy-7,8,2',5'-tetramethoxyflavone 5-O-glucoside	C19H18O7	10.5	-2.34	359.1117	381.0929	344.09, 329.07, 313.07, 298.08, 197.04, 165.02	R, Fl, Fr
66	Flavonoids	25.01	Unknown	C20H20O8	10.5	-1.11	389.1221	411.1033	359.08, 341.07, 328.09, 223.06, 197.04, 183.03, 165.02, 151.04	R, Fl
67	Flavonoids	25.35	Unknown	C19H20O7	9.5	-1.10	361.1271	383.1091	345.09, 319.12, 283.05, 255.05, 223.06, 197.04	R, S, Fl
68(R8)	Flavonoids	25.	**Moslosooflavone**	C17H14O5	10.5	-1.94	299.0	321.0	283.06, 266.06,	R, S, Fl,

		36					908	724	255.07, 238.06, 197.04, 182.02, 171.07	Fr
69	Flavonoids	25.84	Isomer of 5-hydroxy-7,8,2',5'-tetramethoxyflavone 5-O-glucoside	C19H18O7	10.5	-1.73	359.1119	381.0933	344.09, 298.08, 197.04, 183.03, 165.02	R, Fl, Fr
70	Flavonoids	26.11	Andrographin	C18H16O6	10.5	-1.69	329.1014	351.0828	314.08, 299.06, 285.07	R, Fl, Fr
71	Flavonoids	26.63	Isomer of 5-hydroxy-7,8,2',5'-tetramethoxyflavone 5-O-glucoside	C19H18O7	10.5	-1.73	359.1119	381.0935	344.09, 329.07, 298.08, 197.04, 183.03, 165.02	R, S
72	Flavonoids	31.56	Unknown	C16H22O4	5.5	-0.76	279.1583	301.1402	205.09, 149.02	R, L, S, Fl, Fr
73	Phenylpropanoids	16.27	Unknown	C29H32O16	13.5	-1.14	637.1752	659.1571	389.12, 374.10, 359.08, 341.07, 197.04, 183.03, 165.02	R
74	Phenylpropanoids	5.34	Unknown	C17H20O10	7.5	-3.45	385.1095		223.06, 205.05, 185.04	R, L, S, Fl, Fr
75	Phenylpropanoids	5.41	1-O-Sinapoyl-β-D-glucose	C17H22O10	6.5	-3.48	387.1251		225.07, 207.06, 203.05, 189.05	R, L, S, Fr
76(R1)	**Phenylpropanoids**	**6.93**	**Chlorogenic acid**	**C16H18O9**	**7.5**	**-2.76**	**355.101**	**377.083**	**337.09, 163.04, 145.03, 135.04**	R, L, S, Fl, Fr
77(R2)	**Phenylpropanoids**	**9.31**	**Isochlorogenic acid B**	**C25H24O12**	**13.5**	**-4.76**	**517.132**	**539.1135**	**499.12, 337.09, 319.08, 163.04, 145.03**	R, L, S, Fl, Fr

78(R3)	**Phenylpropanoids**	**9.68**	**3,5-Dicaffeoylquinic acid**	**C25H24O12**	**13.5**	**-4.41**	**517.132**	**539.114**	**499.12, 337.09, 319.08, 163.04, 145.03**	R, L, S, Fl, Fr
79	Phenylpropanoids	10.18	Unknown	C25H24O12	13.5	-4.48	517.1315	539.1134	499.12, 337.09, 319.08, 163.04, 145.03	R, L, S, Fl, Fr
80	Phenylpropanoids	10.22	Unknown	C25H26O13	12.5	-2.53	535.1421	557.1421	517.13, 355.10, 337.09, 163.04	R, L, S, Fl, Fr
81	Phenylpropanoids	13.41	Unknown	C26H40O9	6.5	-0.86	497.2737	519.2554	501.18, 357.20, 339.14, 319.08, 179.07, 163.04	R, L, S, Fl, Fr
82	Phenylpropanoids	16.27	Unknown	C26H26O13	13.5	-0.58	547.1441	569.1248	299.09, 284.07, 255.07, 207.03,	R, L, S, Fl, Fr
83	Spermidine derivatives	14.24	Tricoumaroyl spermidine or isomer	C34H37O6N3	17.5	3.94	584.2739	606.2511	438.24, 420.23, 292.20, 275.18, 129.14	L, S, Fl, Fr
84	Spermidine derivatives	14.84	Tricoumaroyl spermidine or isomer	C34H37O6N3	17.5	-3.31	584.2737	606.2535	438.24, 420.23,292.20, 275.18, 129.14	L, S, Fl, Fr
85	Spermidine derivatives	15.05	Dicoumaroyl feruloyl spermidine	C35H39O7N3	17.5	-2.02	614.2841	636.2651	596.28, 468.24, 450.24, 420.23, 305.19, 147.04, 129.14	L, S, Fl, Fr
86	Phosphatidylcholines	26.41	Unknown	C26H50O7NP	3.5	-1.37	518.3228	540.305	500.31, 441.24, 335.26, 258.11, 184.07	R, L, S, Fl, Fr

87	Phosphatidylcholines	27.94	Unknown	C23H44O7NP	2.5	-1.10	478.2917	500.2737	460.28, 337.27, 319.26, 263.24, 198.05	R, L, S, Fl, Fr
88	Phosphatidylcholines	28.08	Unknown	C27H54O12NP	1.5	-1.22	616.3444	638.3265	598.34, 580.33, 532.30, 313.27, 286.07, 268.06, 206.10, 188.09	R, L, S, Fl, Fr
89	Phosphatidylcholines	28.53	1-Linoleoylglycerophosphocholine	C26H50O7NP	2.5	-1.21	520.3386	542.3208	502.33, 443.26, 335.26, 258.11, 184.07	R, L, S, Fl, Fr
90	Phosphatidylcholines	29.06	Unknown	C21H44O7NP	0.5	-0.89	454.2919	476.2737	436.28, 313.27, 249.08, 216.06, 198.05	R, L, S, Fl, Fr
91	Phosphatidylcholines	29.59	1-Palmitoyllysophosphatidylcholine	C24H50O7NP	0.5	-1.42	496.3383	528.3167	478.33, 419.26, 258.11, 184.07	R, L, S, Fl, Fr
92	Phosphatidylcholines	30.61	1-Oleoylglycerophosphocholine	C26H52O7NP	1.5	-1.29	522.3541	544.3354	504.35, 445.27, 417.24, 337.27, 263.24, 184.07	R, L, S, Fl, Fr
93	Phosphatidylcholines	33.38	1-Stearoylglycerophosphocholine	C26H54O7NP	0.5	-0.71	524.3704	546.3525	506.36, 341.31, 282.14, 258.11, 184.07	R, L, S, Fl, Fr
94	Triterpenoids	9.23	Unknown	C33H52O11	7.5	-1.979	625.356	647.3363	463.31, 321.21, 303.20, 285.19, 267.17, 249.16	R, S
95	Triterpenoids	9.36	Unknown	C33H54O11	6.5	-1.509	627.372	649.3546	465.32, 321.21, 303.20, 285.19,	R, S

									267.17, 249.16	
96	Triterpenoids	11.79	Unknown	C33H52O11	7.5	-1.789	625.356	647.3383	463.31, 321.21, 303.20, 285.19, 267.17, 249.16	R, S
97	Triterpenoids	12.2	Unknown	C39H62O13	8.5	-3.038	739.423		577.37, 433.26, 397.31, 379.30, 271.21, 253.20	R, S
98	Triterpenoids	12.33	Unknown	C39H64O13	7.5	-2.268	741.44		579.39, 561.38, 543.37, 435.27, 273.22, 255.21	R, S
99	Triterpenoids	12.53	Unknown	C33H54O11	6.5	-2.309	627.372		465.32, 321.21, 303.20, 285.19, 267.17, 249.16	R, S
100	Triterpenoids	25.59	Toosendanoside+Glc	C33H56O14	5.5	-1.821	677.373	699.3542	515.32, 353.27, 317.25, 279.23, 261.22, 243.21	R, S, L
101	Triterpenoids	27.59	Unknown	C33H58O14	4.5	-4.287	679.387	701.3702	517.33, 355.28, 337.27	L, Fl, Fr
102	Triterpenoids	28.73	Unknown	C33H56O14	5.5	-4.653	677.371		515.32, 353.27, 259.08	R, L, S, Fl, Fr
103	Triterpenoids	29.55	Toosendanoside	C29H44O9	7.5	-1.849	515.32	537.3041	353.27, 335.26, 317.25, 259.08	R, L, S, Fl, Fr
104	Triterpenoids	29.63	Desacylkondurangogenin C 3-O-α-D-glucopyranosyl-(1->4)-O-α-L-fucopyranoside	C33H56O14	5.5	-1.423	677.373		515.32, 353.27	R, S, L
105	Triterpenoids	30.3	Toosendanoside+2H	C27H49O9	3.5	-3.324	517.335	539.3172	355.28, 337.27	R, L, S, Fl, Fr

106	Triterpenoids	30.36	Isomer of desacylkondurangogenin C 3-O-α-D-glucopyranosyl-(1->4)-O-α-L-fucopyranoside	$C_{33}H_{56}O_{14}$	5.5	-1.42	677.373		515.32, 353.27	R, S, L
107	Others	0.96	Agmatine	$C_5H_{13}N_4$	1.5	-2.13	131.1287		114.10, 97.08, 72.08	R, L, S, Fl, Fr
108	Others	1.00	DL-Arginine	$C_6H_{14}N_4O_2$	1.5	-2.69	175.1184	197.1002	158.09, 130.10, 116.07, 70.07	R, L, S, Fl, Fr
109	Others	1.05	Asparagine	$C_4H_8N_2O_3$	1.5	-2.18	133.0606		116.03, 87.06, 70.03	R, L, S, Fl, Fr
110	Others	1.08	L-Glutamine	$C_5H_{10}N_2O_3$	1.5	-0.13	147.0764		130.05, 101.07, 112.05, 102.06, 84.04	R, L, S, Fl, Fr
111	Others	1.23	Proline	$C_5H_9NO_2$	1.5	-2.48	116.0703	138.0546	98.06, 70.07	R, L, S, Fl, Fr
112	Others	5.20	Adenosine	$C_{10}H_{11}N_5O_4$	6.5	-1.32	268.1035		136.06, 115.54, 77.63	R, L, S, Fl, Fr
113	Others	5.62	L-Phenylalanine	$C_9H_{11}NO_2$	4.5	-1.81	166.0858	188.0675	149.06, 120.08, 131.05, 103.05, 93.07	R, L, S, Fr
114	Others	7.33	5,7-Dihydroxy-2-(4-hydroxyphenyl)-6,8-bis[3,4,5-trihydroxy-6-(hydroxymethyl)tetrahydro-2H-pyran-2-yl]-4H-chromen-4-one	$C_{27}H_{30}O_{15}$	12.5	-1.17	595.1662	617.1473	577.16, 457.12, 439.10,409.09, 379.08, 325.07	R, L, S, Fl, Fr
115	Others	8.10	5,7-dihydroxy-2-(4-hydroxyphenyl)-6-[(2S,3R,4R,5S,6R)-3,4,5-trihydroxy-6-(hydroxymethyl)oxan-2-yl]-8-[(2S,3R,4R,5R,6S)-3,4,5-trihydroxy-6-methyloxan-2-yl]-4H-chromen-4-one	$C_{27}H_{30}O_{14}$	12.5	-2.00	579.1695	601.1504	561.16, 495.13, 441.12, 393.10, 363.09, 321.08, 309.08	R, L, S
116	Others	8.61	2-(3,4-Dihydroxyphenyl)-5-hydroxy-4-oxo-4H-chromen-7-yl 6-O-(6-deoxy-alpha-L-mannopyranosyl)-beta-D-glucopyranoside	$C_{27}H_{30}O_{15}$	12.5	-1.20	595.1652	617.1466	449.11, 287.06	L, Fl, Fr
117	Others	9.5	5-hydroxy-3-(4-hydroxyphenyl)-7-{[(2S,3R,4S,5S,6R)-3,4,5-trihydroxy-6-({[(2R,3R,4R,5R,6S)-3,4,5-trihydroxy-6-	$C_{27}H_{30}O_{14}$	12.5	-1.67	579.1	601.1	433.11, 271.06	L, S, Fl,

		4	methyloxan-2-yl]oxy}methyl)oxan-2-yl]oxy}-4H-chromen-4-one				701	507		Fr
118	Others	9.73	Tricin 5-O-b-D-glucoside	C23H24O12	11.5	-0.80	493.1328	517.1322	331.08, 316.06, 298.05, 287.06	R, S, Fl
119	Others	10.23	5-hydroxy-2-(4-hydroxyphenyl)-6-methoxy-7-{[(2S,3R,4S,5S,6R)-3,4,5-trihydroxy-6-(hydroxymethyl) oxan-2-yl]oxy}-4H-chromen-4-one	C22H22O11	11.5	-1.11	463.1233	485.1046	301.07, 259.06, 167.03, 135.04	R
120	Others	11.04	(2b,3b,5b,20xi,22R)-2,3,14,20,22,25-Hexahydroxy-6-oxocholest-7-en-26-al	C27H42O8	6.5	1.12	495.2958	517.1691	477.29, 349.26, 275.18, 204.10	L, S, Fl, Fr
121	Others	12.24	1-Stearyl estercitric acid	C24H44O7	2.5	4.31	445.3179	467.3001	392.27, 350.25, 222.15, 205.12, 162.12	L, S, Fl, Fr
122	Others	16.73	{6-[5,7-Dihydroxy-2-(4-hydroxyphenyl)-4-oxo-6-(3,4,5-trihydroxytetrahydro-2H-pyran-2-yl)-4H-chromen-8-yl]-3,4,5-trihydroxytetrahydro-2H-pyran-2-yl} methyl acetate	C28H30O15	13.5	-1.72	607.1647		589.16, 571.15, 359.11, 344.09, 311.06, 298.08	L, S, Fl, Fr
123	Others	16.95	1,5-Anhydro-6-O-(4-carboxy-3-hydroxy-3-methylbutanoyl)-1-[5,7-dihydroxy-2-(4-hydroxyphenyl)-4-oxo-4H-chromen-8-yl] hexitol	C27H28O14	13.5	-1.53	577.1543	599.1346	559.15, 329.10, 314.08, 299.06, 285.08, 165.02	R, S
124	Others	19.28	2-(2,6-dimethoxyphenyl)-5,6-dimethoxy-4H-chromen-4-one	C19H18O6	10.5	-0.80	343.1173	365.0987	328.10, 313.07, 299.09, 285.08, 197.04, 181.01	R, L, S, Fl, Fr
125	Others	22.31	Glucosylphytosphingosin	C24H49NO8	0.5	-0.58	480.3525	502.3345	462.34, 444.33, 426.32, 384.31, 342.30, 300.29, 206.10	R, L, S, Fl, Fr
126	Others	23.26	2-Amino-1,3,4-octadecanetriol	C18 H39NO3	0	-1.41	318.2997	340.2812	300.29, 282.28, 270.28, 264.27, 240.27	R, L, S, Fl, Fr

Bold indicates the components were compared with reference compounds. R: the number of reference compounds in figure. 1.

Table S4 Components with VIP > 3 between different harvest periods and different plant parts of *A. paniculata* based on the OPLS-DA.

NO.	Components	VIP	*p*	NO.	Components	VIP	*p*
1(R11)	Andrographolide	16.65	$2.19*10^{-17}$	22	10.95_641.33m/z	4.09	$2.41*10^{-28}$
2(R18)	Dehydroandrographolide	12.34	$2.49*10^{-13}$	23	24.45_301.11m/z	4.06	$1.11*10^{-12}$
3(R14)	14-Deoxyandrographolide	11.95	$2.68*10^{-19}$	24	16.85_329.1013m/z	3.90	$4.74*10^{-11}$
4	15.69_549.16m/z	8.82	$9.22*10^{-11}$	25	18.16_301.22m/z	3.90	$1.17*10^{-9}$
5	13.34_497.27m/z	7.50	$9.03*10^{-16}$	26	21.47_375.11m/z	3.89	$4.17*10^{-11}$
6	16.83_577.15m/z	6.86	$1.67*10^{-19}$	27	15.21_746.36m/z	3.86	$1.23*10^{-44}$
7	25.83_389.12m/z	6.79	$1.33*10^{-16}$	28	26.68_389.12m/z	3.84	$4.83*10^{-16}$
8	24.78_359.11m/z	6.51	$9.89*10^{-15}$	29	15.02_614.28m/z	3.83	$4.09*10^{-38}$
9	10.15_447.09m/z	5.82	$1.68*10^{-28}$	30	15.06_491.15m/z	3.82	$2.45*10^{-19}$
10	14.74_551.17m/z	5.36	$2.66*10^{-45}$	31	12.76_477.14m/z	3.80	$1.62*10^{-10}$
11	Andrographidine B	5.24	$1.55*10^{-18}$	32	12.70_517.17m/z	3.73	$4.35*10^{-26}$
12	15.08_813.40m/z	4.97	$3.77*10^{-9}$	33	16.25_637.17m/z	3.71	$3.22*10^{-10}$
13	14.37_521.16m/z	4.89	$3.87*10^{-16}$	34	15.13_398.19m/z	3.68	$2.69*10^{-14}$
14	26.14_389.12m/z	4.86	$7.45*10^{-13}$	35	9.66_517.13m/z	3.67	$4.99*10^{-12}$
15	20.23_333.20m/z	4.83	$2.17*10^{-14}$	36	18.13_584.31m/z	3.65	$7.04*10^{-10}$
16	15.13_407.21m/z	4.70	$6.93*10^{-11}$	37	Andrographidine A	3.63	$2.61*10^{-21}$
17	6.72_595.16m/z	4.69	$2.3*10^{-76}$	38	14.85_563.14m/z	3.60	$1.54*10^{-12}$
18	15.31_593.15m/z	4.46	$2.72*10^{-13}$	39	6.69_463.33m/z	3.53	$1.77*10^{-9}$
19	16.83_481.28m/z	4.36	$2.68*10^{-27}$	40	15.36_521.16m/z	3.52	$5.17*10^{-15}$
20	11.42_440.23m/z	4.18	$1.32*10^{-10}$	41	16.63_359.11m/z	3.50	$1.10*10^{-9}$
21	14.37_299.09m/z	4.10	$5.93*10^{-18}$				

R: the number of reference compounds in figure. 1.
